# A comparison of trends in the incidence of non-alcoholic steatohepatitis-related liver cancer in the BRICS countries from 1990 to 2021, alongside projections for the next 15 years

**DOI:** 10.3389/fonc.2025.1632843

**Published:** 2025-09-15

**Authors:** Congjie Chen, Siying Huang, Huiqiang Wu, Weitao Hu, Chaowei Li, Dongwei Du, Taiyong Fang

**Affiliations:** ^1^ Department of Gastroenterology, the Second Affiliated Hospital, Fujian Medical University, Quanzhou, Fujian, China; ^2^ Department of Hematopathology, the Second Affiliated Hospital, Fujian Medical University, Quanzhou, Fujian, China

**Keywords:** BRICS countries, non-alcoholic steatohepatitis-associated hepatocellular carcinoma, incidence, prediction, trend

## Abstract

**Background:**

Despite the ongoing rise in the global burden of non-alcoholic steatohepatitis-related liver cancer (NALC), systematic analyses and long-term trend projections of the disease’s burden in the BRICS countries (Brazil, Russia Federation, India, China, and South Africa) remain relatively scarce.

**Objective:**

The objective of this study was to systematically assess the incidence dynamics of NALC in the BRICS countries during the period from 1990 to 2021 to reveal its epidemiological characteristics, to assess the potential public health challenges, and to forecast the development trends over the next 15 years.

**Methods:**

The present study collated and systematized the incidence data of NALC in the BRICS countries from 1990 to 2021, drawing upon the global burden of disease database (2021). The indicators that were analyzed included the incidence rate, the age-standardized incidence rate, the annual percentage change, and the average annual percentage change. The analysis incorporated the utilization of two distinct models: the joinpoint regression model and the age–period–cohort model. These models were employed to evaluate the temporal effects and population characteristics. Additionally, the autoregressive integrated moving average model was implemented to offer insights into the potential future risk of the disease.

**Results:**

From 1990 to 2021, the incidence of NALC in the BRICS countries demonstrated a marked increase. The incidence rate in China exhibited an increase from 0.34 (95% uncertainty interval (UI): 0.28–0.42) to 0.79 (95% UI: 0.61–1.01). Brazil demonstrated a rise from 0.07 (95% UI: 0.06–0.08) to 0.17 (95% UI: 0.15–0.20). The Russian Federation demonstrated a rise from 0.14 (95% UI: 0.12–0.16) to 0.39 (95% UI: 0.33–0.45). India demonstrated a rise from 0.13 (95% UI: 0.10–0.16) to 0.32 (95% UI: 0.26–0.38). South Africa demonstrated a rise from 0.29 (95% UI: 0.18–0.47) to 0.74 (95% UI: 0.59–0.89). The sex analysis demonstrated that, in the majority of countries except India, the male incidence rate exhibited a higher rate of increase than the female incidence rate. The steepest age-specific increase occurred in the oldest cohorts, notably 90+ years. The predictions derived from the autoregressive integrated moving average model indicate that the prevalence of NALC morbidity is anticipated to escalate in the BRICS nations over the ensuing 15-year period.

**Conclusion:**

The present study reveals a sustained upward trend in NALC incidence in the BRICS countries during 1990–2021 and significant differences in the pace and drivers of growth across countries. The heterogeneity reflected by the joinpoint and APC models reveals gaps in the burden of metabolic diseases, public health response, and policy implementation across countries. In order to address this challenge, priority should be given to the promotion of metabolic disease-related early screening, health behavior interventions, and systematic management.

## 1 Introduction

Non-alcoholic fatty liver disease (NAFLD) is a prevalent chronic liver disease that is frequently linked to metabolic abnormalities, including obesity, insulin resistance, type 2 diabetes mellitus, hypertension, hyperlipidemia, and metabolic syndrome (1, 2). The pathological progression of NAFLD is characterized by a series of stages, ranging from non-alcoholic fatty liver to non-alcoholic steatohepatitis (NASH). Of these, NASH is considered to be the key link in the progression of liver fibrosis, cirrhosis, and ultimately hepatocellular carcinoma (HCC) (3). The recent years have seen a global increase in the incidence of non-alcoholic steatohepatitis-related liver cancer (NALC). This phenomenon is projected to exert a considerable pressure on public health systems and the economy in the forthcoming decades (4).

Brazil, Russia Federation, India, China, and South Africa (BRICS) are of pivotal significance in terms of population size, socio-economic structure, and global health landscape (5). In comparison with high-income countries, the BRICS countries are undergoing a rapid and complex socio-economic transition. This is characterized by accelerated urbanization, ageing populations, Westernized lifestyle, and an uneven distribution of healthcare resources (6). These structural changes are likely to have a considerable impact on the onset and progression of NALC. A systematic study of the prevalence trends of NALC in the BRICS countries is required to facilitate a comprehensive understanding of the influence of urbanization, disparities in basic health services, and socio-economic inequalities on the evolution of metabolic diseases within specific cultural and institutional contexts (7).

The Global Burden of Disease (GBD) 2021 study provides a comprehensive and up-to-date dataset, which forms a key basis to assess the burden of disease, temporal trends, and geographic variation in NALC (8). The database integrates multi-source data from all regions of the world, with a focus on epidemiological indicators of incidence and mortality, along with their main risk factors (8–10). The systematic methodology of GBD 2021 is highly standardized and comparable, ensuring the accuracy and consistency of the estimation results across different regions and time periods. Consequently, it is widely used for global and regional disease trend studies (1). Despite the fact that studies have analyzed the global prevalence trends of NALC based on data from 1990 to 2019, there is a paucity of in-depth exploration of the significant heterogeneity and local variations within the BRICS countries (11). Consequently, detailed epidemiological analyses of these countries and the development of targeted public health intervention strategies are imperative to alleviate the growing burden of NALC.

The present study constitutes a systematic analysis of the trend of NALC incidence in the BRICS countries from 1990 to 2021. Joinpoint regression model was utilized in order to identify overall trends in incidence rates and potential inflection points during the specified period (12). This approach was then combined with an age–period–cohort model to assess the independent effects of age, period, and birth cohort on the incidence of NALC (13). Furthermore, the autoregressive integrated moving average (ARIMA) model was employed to predict the incidence trend over the following 15 years (14). The objective of this study is to facilitate the formulation of scientific and precise public health intervention strategies in the BRICS countries.

## 2 Methods

### 2.1 Data sources

Data for this study were obtained from the GBD 2021 public database, which is available through the GBD results query tool (http://ghdx.healthdata.org/gbd-results-tool) in the global health data exchange (15). The GBD 2021 system assesses the burden of disease for a total of 371 diseases and injuries in 204 countries and territories around the world, covering a range of key health indicators such as incidence rates, years of life lost, and disability-adjusted life years (8–10, 16). In the present study, NALC-related morbidity data was extracted from the BRICS countries (Brazil, the Russian Federation, India, China, and South Africa) from 1990 to 2021. The analysis was conducted on the overall trend and stage change characteristics of NALC, with incidence rate (IR) and age-standardized incidence rate (ASIR) serving as the primary analytical indicators. Among them, IR indicates the number of new cases per 100,000 person-years in the study population; ASIR is age-structured using the world’s standard population, which allows comparable analyses in different populations or between time periods and avoids the effect of differences in age distribution. The objective of the study was to elucidate the long-term evolution of the disease across diverse geographical regions and demographic groups. All incidence rates are expressed per 1,000,000 reported population.

The present study utilized the age–period–cohort model, drawing upon the GBD 2021 database, to analyze the trend of NALC incidence in the BRICS countries from 1990 to 2021. Meanwhile, the joinpoint regression model was utilized to identify the inflection points of incidence rate changes in different periods. Furthermore, the ARIMA model was employed to predict the incidence trend of NALC in the subsequent 15 years. In order to estimate 95% uncertainty interval (UI), this study conducted 1,000 replicate samples. These were based on the model selection, parameter estimation, and data quality control strategies employed in the GBD modeling process. Uncertainty intervals were constructed by taking the 2.5th and 97.5th percentile of the simulation results (17). All data were obtained from publicly available databases, and approval for exemption from informed consent was obtained from the University of Washington Institutional Review Board.

### 2.2 Joinpoint regression analysis

Joinpoint regression is a statistical method employed for the analysis of the dynamics of morbidity. This is achieved by identifying multiple trend inflection points in a time series (18). This approach is more sensitive to changes in data structure than single trend fitting (e.g., Poisson regression or general time series modeling) (18). The model is capable of identifying key turning points by detecting time points of significant trend changes and calculating the annual percentage change (APC) for each phase (19). The resulting outputs include the APC at each stage and the overall average annual rate of change (AAPC). Differences between countries or populations are assessed by comparing the AAPC and its confidence interval (CI). Statistical significance was determined at a two-sided *P <*0.05.

### 2.3 Age–period–cohort analysis

The age–period–cohort model has been used to explain long-term changes in the number of cases of disease over time (20). The estimation of independent effects of factors is complicated by the inherent identification difficulties associated with the strict linear relationship between age, period, and birth cohort (birth cohort = period − age) (21). In the present study, the age–period–cohort model was employed to decompose the IR into an age effect, a period effect, and a birth cohort effect, with a view to explore the relative contributions of these three factors to changes in NALC incidence (22).

To satisfy the model’s framework, we extracted the rates from the GBD data and divided them into successive 5-year groups (15–19, 20–24,…, 95+ years) and consecutive 5-year periods from 1990 to 2021 (23). We also divided them into consecutive 5-year birth cohort groups, starting from 1897–1901 to 2017–2021 (24). In each time period, the number of NALC incidence cases and the corresponding cumulative IR were calculated for each age group. The National Cancer Institute’s web-based age–period–cohort tool was utilized for the purpose of conducting analyses pertaining to age period and cohort (14). The analysis was conducted using the online tool (https://analysistools.cancer.gov/apc/), with additional data visualization and statistical analysis performed in R (version 4.2.3) (25, 26). It is imperative to note that all hypothesis tests were two-sided, and a *P*-value less than 0.05 was deemed to be statistically significant (20).

### 2.4 Forecasting the burden of NALC in the BRICS countries from 2022 to 2036

The ARIMA model is a well-known way of predicting future trends. It can effectively capture trends, seasonality, and random volatility characteristics in the data by combining three components: autoregression, difference, and moving average (27). In this study, we used a special type of computer program (called the ARIMA model) to try and predict how the NALC problem will develop in the BRICS countries from 2022 to 2036.

## 3 Results

### 3.1 Trends in NALC incidence in the BRICS countries from 1992 to 2021

**Figure 1:**
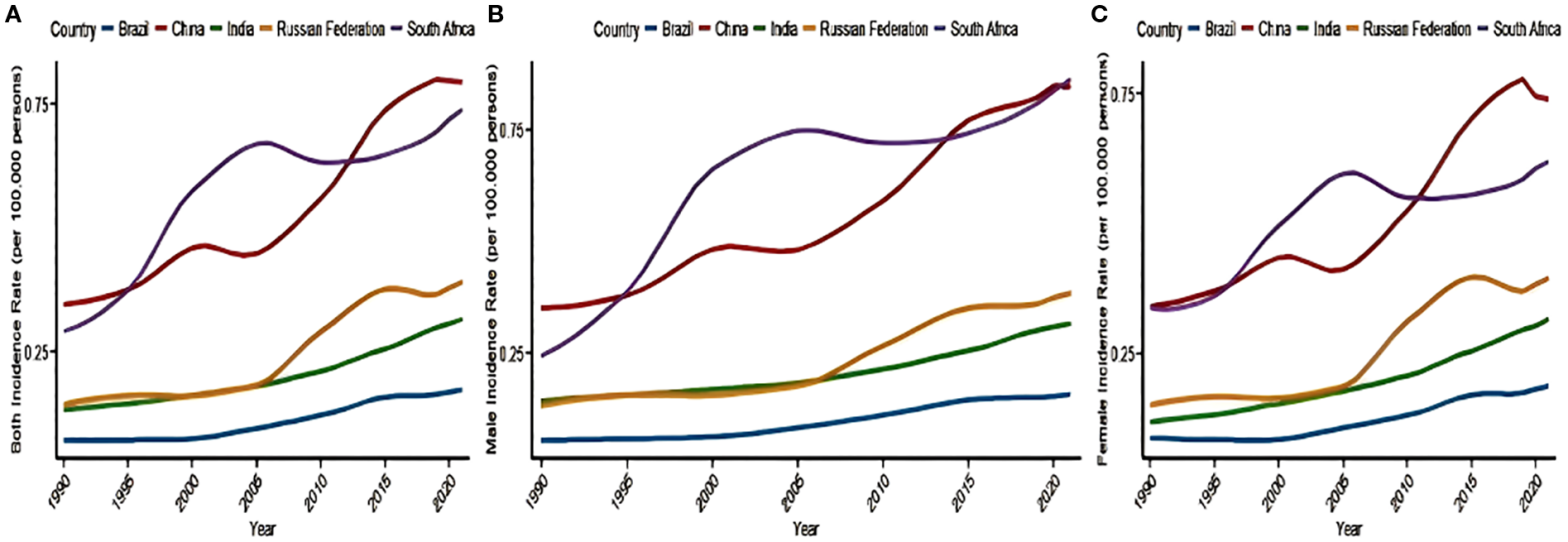
Trends in the incidence of NALC in the BRICS countries—**(A)** both sex, **(B)** female, and **(C)** male)—from 1990 to 2021. NALC, non-alcoholic steatohepatitis-related liver cancer.

**Tables 1** and **2** and **Figure 1** illustrate the trend of NALC IR for the BRICS countries (Brazil, Russian Federation, India, China, and South Africa) during the period from 1990 to 2021. The collective IR of the BRICS countries demonstrated an escalating tendency over the observed period. The Russia Federation and South Africa had the highest rate of increase in the number of cases, and Brazil had the lowest. The Russian Federation IR increased from 0.14 (95% UI: 0.12–0.16) to 0.39 (95% UI: 0.33–0.45), with the highest recorded in the 90–94 years age group (6.55, 95% UI: 4.56–8.75). In South Africa, the IR increased from 0.29 (95% UI: 0.18–0.47) to 0.74 (95% UI: 0.59–0.89), with the highest recorded in ages 95 years and above (15.15, 95% UI: 9.47–21.83). In China, the IR increased from 0.34 (95% UI: 0.28–0.42) in 1990 to 0.79 (95% UI: 0.61–1.01) in 2021, with the highest recorded in the 85–89 years age group (6.55, 95% UI: 4.56–8.75). The India IR increased from 0.13 (95% UI: 0.10–0.16) to 0.32 (95% UI: 0.26–0.38), with the highest prevalence recorded in the 80–84 years age group (3.45, 95% UI: 2.60–4.33). In Brazil, the IR increased from 0.07 (95% UI: 0.06–0.08) to 0.17 (95% UI: 0.15–0.20), with the highest recorded in the 85–89 years age group (1.64, 95% UI: 1.16–2.13).

**Table 1:** Incidence and average annual percentage change of NALC in the BRICS countries in 1990 and 2021.

Categories	Sex	Rate in 1990, 95% UI	Rate in 2021, 95% UI	*P*-value	AAPC-95CI
Global					
Brazil	Both	0.07 (0.06, 0.08)	0.17 (0.15, 0.2)	*P* < 0.001	0.81 (0.67–0.96)
	Female	0.09 (0.08, 0.1)	0.19 (0.16, 0.22)	*P* < 0.001	0.36 (0.16–0.56)
	Male	0.05 (0.05, 0.06)	0.16 (0.13, 0.18)	*P* < 0.001	1.43 (1.34–1.52)
Russia Federation	Both	0.14 (0.12, 0.16)	0.39 (0.33, 0.45)	*P* < 0.001	2.11 (1.59–2.64)
	Female	0.15 (0.13, 0.18)	0.39 (0.33, 0.46)	*P* < 0.001	2.02 (1.40–2.64)
	Male	0.13 (0.11, 0.15)	0.38 (0.32, 0.46)	*P* < 0.001	2.00 (1.67–2.32)
India	Both	0.13 (0.1, 0.16)	0.32 (0.26, 0.38)	*P* < 0.001	1.50 (1.42–1.58)
	Female	0.12 (0.09, 0.14)	0.31 (0.26, 0.38)	*P* < 0.001	1.66 (1.60–1.72)
	Male	0.14 (0.11, 0.17)	0.32 (0.26, 0.39)	*P* < 0.001	1.39 (1.33–1.45)
China	Both	0.34 (0.28, 0.42)	0.79 (0.61, 1.01)	*P* < 0.001	0.44 (0.34–0.54)
	Female	0.34 (0.26, 0.42)	0.74 (0.56, 0.98)	*P* < 0.001	0.14 (0.03–0.24)
	Male	0.35 (0.27, 0.45)	0.85 (0.6, 1.16)	*P* < 0.001	0.76 (0.66–0.87)
South Africa	Both	0.29 (0.18, 0.47)	0.74 (0.59, 0.89)	*P* < 0.001	2.10 (1.88–2.31)
	Female	0.34 (0.23, 0.51)	0.62 (0.52, 0.74)	*P* < 0.001	0.99 (0.85–1.13)
	Male	0.24 (0.1, 0.49)	0.86 (0.66, 1.11)	*P* < 0.001	3.32 (3.10–3.55)

UI, uncertainty interval; CI, confidence interval; AAPC, average annual percentage change; IR, incidence rate; NALC, nonalcoholic steatohepatitis-associated liver cancer.

### 3.2 Joinpoint regression analysis

**Figure 2:**
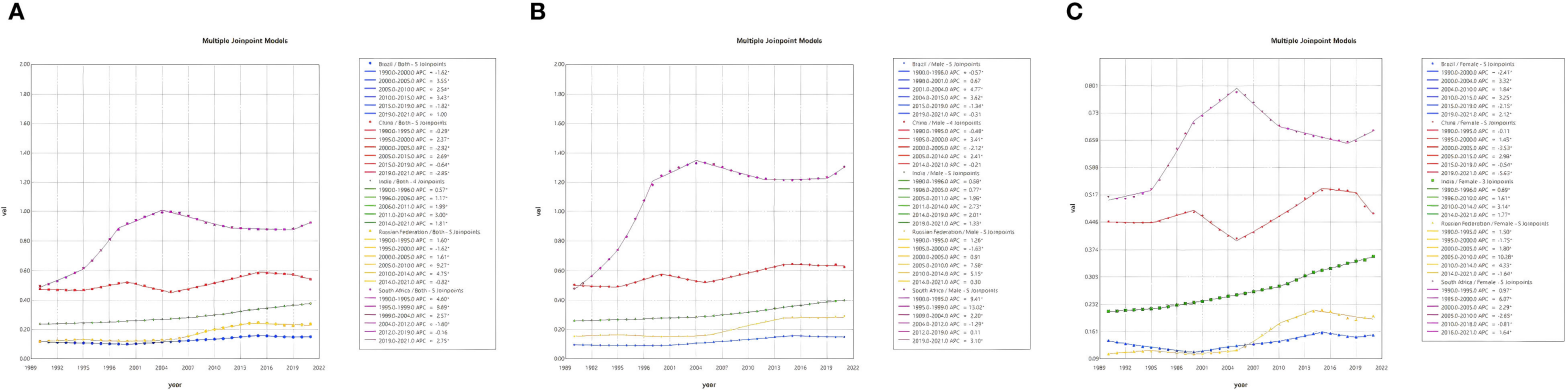
Joinpoint regression analysis of NALC incidence rate in the BRICS countries from 1990 to 2021 [both sex **(A)**, male **(B)**, and female **(C)**].

As illustrated in **Table 2**; **Figure 2**, an investigation into the annual percentage change in NALC ASIR and AAPC for the BRICS countries from 1990 to 2021 was conducted in order to assess their trends over time. A sex-stratified analysis revealed that the ASIR was generally higher in men than in women, with the exception of India, where the opposite trend was observed, with faster ASIR growth in women [adjusted average percentage change (AAPC): 1.66 (95% confidence interval [CI]: 1.60–1.72) vs. 1.39 (95% CI: 1.33–1.45)]. Connected-point regression analysis demonstrates that the variation in ASIR across countries is characterized by non-linear phases. The period under consideration saw South Africa’s ASIR reaching its zenith in 2004; China’s rapid growth in the early years was followed by a gradual slowdown thereafter; the Russian Federation’s marked increase from 2000 onward was followed by stabilization after 2010; India’s overall growth was sustained, at a lower level than that of China and South Africa; and Brazil was relatively stable, with only minor fluctuations.

**Table 2:** The rates and average annual percentage change of NALC in different age group from 1990 to 2021 in the BRICS countries.

Categories	Brazil		China		India		Russian Federation		South Africa	
	Rates in 2021, 95% UI	AAPC_95CI	Rates in 2021, 95% UI	AAPC_95CI	Rates in 2021, 95% UI	AAPC_95CI	Rates in 2021, 95% UI	AAPC_95CI	Rates in 2021, 95% UI	AAPC_95CI
15–19	0.01 (0.01,0.01)	0.77 (1.05 - 1.00)	0.01 (0.01,0.02)	-0.74 (-0.44 - 1.00)	0.02 (0.01,0.03)	0.01 (0.37 - 1.00)	0.02 (0.02,0.03)	-0.22 (0.61 - 0.00)	0.03 (0.02,0.04)	-0.03 (1.13 - 0.00)
20–24	0.01 (0.01,0.01)	0.98 (1.17 - 1.00)	0.02 (0.01,0.03)	-0.88 (-0.14 - 1.00)	0.02 (0.02,0.03)	0.45 (1.29 - 1.00)	0.02 (0.01,0.02)	1.02 (1.31 - 1.00)	0.05 (0.04,0.07)	-0.45 (0.32 - 0.00)
25–29	0.01 (0.01,0.02)	-0.17 (0.32 - 0.00)	0.05 (0.03,0.07)	-0.53 (0.22 - 0.00)	0.03 (0.02,0.04)	1.07 (1.50 - 1.00)	0.02 (0.01,0.03)	1.92 (2.83 - 1.00)	0.1 (0.07,0.15)	-1.22 (-0.15 - 1.00)
30–34	0.02 (0.01,0.02)	-0.15 (0.08 - 0.00)	0.09 (0.06,0.13)	-0.55 (0.12 - 0.00)	0.04 (0.03,0.06)	1.13 (1.76 - 1.00)	0.03 (0.02,0.04)	1.89 (3.46 - 1.00)	0.22 (0.16,0.29)	-0.64 (0.01 - 0.00)
35–39	0.03 (0.02,0.04)	-0.20 (0.15 - 0.00)	0.17 (0.12,0.24)	-0.32 (0.24 - 0.00)	0.07 (0.05,0.1)	1.24 (1.58 - 1.00)	0.04 (0.03,0.06)	1.15 (2.80 - 1.00)	0.36 (0.24,0.49)	-0.09 (0.86 - 0.00)
40–44	0.05 (0.04,0.07)	-0.16 (0.11 - 0.00)	0.32 (0.22,0.46)	-0.55 (-0.00 - 1.00)	0.13 (0.09,0.17)	1.12 (1.45 - 1.00)	0.09 (0.06,0.11)	1.04 (2.42 - 1.00)	0.51 (0.36,0.71)	0.73 (1.06 - 1.00)
45–49	0.09 (0.07,0.12)	-0.15 (0.07 - 0.00)	0.55 (0.38,0.79)	-0.35 (0.11 - 0.00)	0.25 (0.18,0.33)	1.06 (1.20 - 1.00)	0.17 (0.12,0.22)	1.07 (2.49 - 1.00)	0.7 (0.49,0.96)	1.03 (1.28 - 1.00)
50–54	0.2 (0.15,0.25)	0.34 (0.58 - 1.00)	0.84 (0.58,1.18)	-0.05 (0.38 - 0.00)	0.46 (0.33,0.6)	0.62 (1.03 - 1.00)	0.29 (0.22,0.38)	1.30 (1.85 - 1.00)	1.38 (0.95,1.86)	1.79 (2.65 - 1.00)
55–59	0.34 (0.25,0.45)	0.94 (1.16 - 1.00)	1.09 (0.72,1.59)	-0.32 (0.07 - 0.00)	0.92 (0.67,1.22)	1.10 (1.46 - 1.00)	0.56 (0.4,0.74)	1.51 (2.59 - 1.00)	2.08 (1.48,2.82)	2.03 (2.65 - 1.00)
60–64	0.54 (0.4,0.7)	1.00 (1.22 - 1.00)	1.74 (1.15,2.54)	0.03 (0.41 - 1.00)	1.34 (0.97,1.78)	0.90 (1.11 - 1.00)	0.79 (0.58,1.06)	1.24 (2.17 - 1.00)	2.61 (1.86,3.62)	2.23 (2.89 - 1.00)
65–69	0.72 (0.53,0.92)	0.84 (1.36 - 1.00)	2.4 (1.62,3.34)	0.13 (0.75 - 1.00)	1.99 (1.46,2.55)	1.10 (1.73 - 1.00)	1.16 (0.86,1.49)	1.18 (2.77 - 1.00)	3.75 (2.69,5.06)	2.28 (2.67 - 1.00)
70–74	0.92 (0.69,1.17)	0.48 (1.13 - 1.00)	3.02 (2.08,4.08)	0.26 (0.67 - 1.00)	2.52 (1.9,3.19)	1.17 (1.56 - 1.00)	1.14 (0.85,1.46)	1.18 (1.73 - 1.00)	4.65 (3.39,6.09)	2.27 (2.62 - 1.00)
75–79	1.24 (0.93,1.61)	0.31 (0.96 - 1.00)	3.76 (2.64,5.11)	0.59 (0.82 - 1.00)	3.04 (2.35,3.88)	1.55 (1.78 - 1.00)	2.13 (1.63,2.76)	2.37 (3.42 - 1.00)	6.54 (4.97,8.73)	2.24 (2.63 - 1.00)
80–84	1.51 (1.09,1.98)	0.27 (0.68 - 1.00)	5.2 (3.62,7.18)	1.73 (2.27 - 1.00)	3.45 (2.6,4.33)	2.09 (2.36 - 1.00)	2.58 (1.9,3.36)	2.68 (3.73 - 1.00)	9.85 (7.16,13.39)	1.88 (2.24 - 1.00)
85–89	1.64 (1.16,2.13)	0.79 (1.20 - 1.00)	6.55 (4.56,8.75)	1.19 (1.51 - 1.00)	3.12 (2.34,4)	2.96 (3.48 - 1.00)	2.51 (1.85,3.21)	3.30 (3.55 - 1.00)	12.17 (8.97,15.69)	2.24 (2.60 - 1.00)
90–94	1.62 (1.09,2.25)	1.00 (1.43 - 1.00)	5.97 (3.86,8.38)	0.99 (1.61 - 1.00)	3 (2.17,3.96)	5.01 (5.77 - 1.00)	2.97 (2.06,4.02)	3.62 (3.80 - 1.00)	13.72 (9.42,18.87)	2.42 (2.80 - 1.00)
95 plus	1.32 (0.78,2.02)	0.39 (0.72 - 1.00)	3.71 (2.2,5.76)	1.83 (3.22 - 1.00)	1.43 (0.94,2.14)	6.93 (7.36 - 1.00)	2 (1.24,2.89)	2.12 (2.74 - 1.00)	15.15 (9.47,21.83)	3.38 (3.75 - 1.00)

UI, uncertainty interval; CI, confidence interval; AAPC, average annual percentage change; IR, incidence rate; NALC, nonalcoholic steatohepatitis-associated liver cancer.

As illustrated in **Figure 2**, joinpoint regression analyses demonstrated that the ASIR of NALC in the BRICS countries exhibited nonlinear variations over the period 1990–2021, with multiple pivotal inflection points, and demonstrated an overall phase increase. Brazil witnessed substantial rises in the period 2000–2005 (APC = 3.55, 95% UI: 3.15–3.94) and 2010–2015 (APC = 3.43, 95% UI: 3.04–3.82), with a notable deceleration occurring after 2015. It is evident that the growth rate underwent a substantial decline after the year 2015. The People’s Republic of China experienced a period of rapid economic growth between 2005 and 2015, with an APC of 2.69 (95% confidence interval: 2.62–2.76). However, this growth trajectory subsequently decelerated. India continued to demonstrate an upward trend, with a period of accelerated growth from 2010 to 2014 (APC = 3.00, 95% UI: 2.32–3.69). The Russian Federation demonstrated a marked increase between 2005 and 2010 (APC = 9.27, 95% UI: 7.68–10.88), subsequently entering a period of relative stability. The South African economy demonstrated a consistent growth from 1990 to 2005, after which it experienced a slowdown, a trend that has persisted since 2010.

### 3.3 Effect of age, period, and cohort on the incidence of NALC

**Figure 3:**
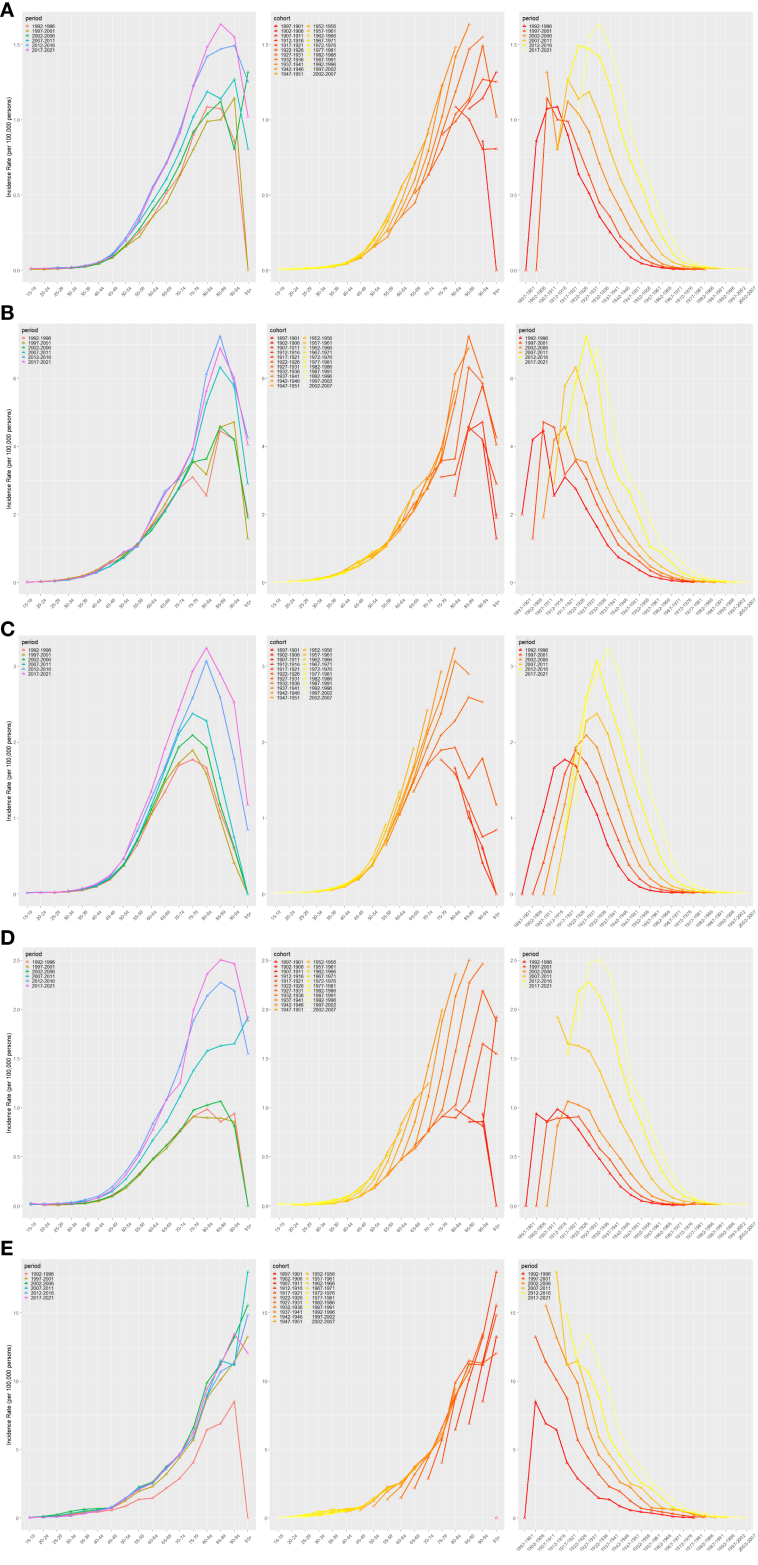
Age–period–cohort model analysis for BRICS IR [Brazil **(A)**, China **(B)**, India **(C)**, Russian Federation **(D)**, and South Africa **(E)**].

The results of the age–period–birth cohort modeling of NALC in the BRICS countries are displayed in **Figure 3**. This analysis reveals the evolutionary trend of NALC incidence and its potential drivers across three dimensions: the age effect, the period effect, and the birth cohort effect. The age effect showed a gradual increase in NALC incidence with age. The steepest age-specific increase occurred in the oldest cohorts, notably 90+ years. It is noteworthy that the incidence of the disease in young adults aged 20–40 years has also been on the rise in recent years, suggesting an increase in the proportion of early-onset cases. Time-effects analysis demonstrated that the population-wide morbidity rates exhibited an upward trend between 1992 and 2010; although certain age groups demonstrated stabilization after 2015, the overall burden of disease remained high. A thorough analysis of birth cohorts reveals an escalating risk of morbidity across generations, with the comparatively lowest rates observed in the 1940–1970 birth cohort. However, a notable surge in risk is evident in the 1980 and subsequent birth cohorts, particularly in the 2000–2007 birth cohort, which exhibits the highest rates.

### 3.4 Projections of the ASIR of NALC in the BRICS countries over the next 15 years

**Figure 4:**
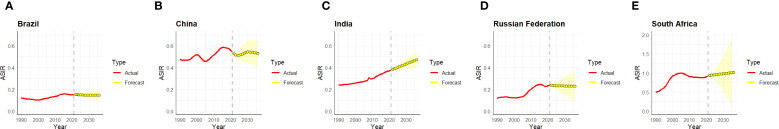
ARIMA model analysis of ASIR in the BRICS countries [Brazil **(A)**, China **(B)**, India **(C)**, Russian Federation **(D)**, and South Africa **(E)**]. ASIR, age-standardized incidence rate; ARIMA, autoregressive integrated moving average.

In order to assess future trends, the present study employed the ARIMA model to forecast the ASIR of NALC in the BRICS countries, the results of which are detailed in **Figure 4**. The findings indicated that the disease burden of NALC in the BRICS countries is anticipated to persist over the ensuing 15-year period, with the ASIR potentially escalating further in certain nations. Among the countries under scrutiny, the projected ASIR in South Africa and India demonstrates a persistent upward trend, with the most substantial increase predicted to exceed 2.0/100,000 after 2030. Conversely, the ASIR projections for China, Brazil, and the Russian Federation demonstrate relatively flat trends and have not undergone significant increases. While China’s ASIR is at a high level, it is expected to stabilize in the future; and the projections for Brazil and the Russian Federation have remained low overall and have not shown a significant upward trend.

## 4 Discussion

In the period from 1990 to 2021, the ASIR of NALC in the BRICS countries (Brazil, the Russian Federation, India, China, and South Africa) exhibited an overall upward trend. However, there were substantial variations in the magnitude and configuration of the changes in incidence rates among the countries. In China, the IR of NALC has continued to increase with accelerated urbanization, rising prevalence of metabolic syndrome, “westernization” of lifestyle, and an aging population (6). Notwithstanding the enhancement of primary care provisions and the incremental progression in chronic disease prevention and control capabilities in recent years, NASH, as a pernicious cause of HCC, has not been the focus of sufficient attention, consequently leading to delayed early identification and intervention (28, 29). The growth of IR in India is relatively gradual, and the overall incidence level is low. However, it is possible that the actual burden may be underestimated. This phenomenon is closely related to two interrelated factors. Firstly, there is an inadequate tumor registry system. Secondly, there is a lack of public awareness of NASH (30).

Recent studies have indicated a marginal increase in the incidence of NALC in Brazil. Brazil’s relatively stable NALC trends likely reflect its long-standing investments in universal health coverage through the Sistema Único de Saúde (SUS), which has expanded access to primary care, hepatitis B vaccination, and chronic disease management programs (31, 32). By contrast, China and India experienced more significant fluctuations in incidence rates, potentially due to variations in healthcare resource allocation between urban and rural areas as well as the ongoing development of the chronic disease prevention and control system (33, 34). Meanwhile, Russia and South Africa exhibited unstable incidence trends due to the prevalence of high-risk behaviors (e.g., tobacco use, alcohol consumption, and obesity), coupled with the delayed implementation of chronic disease management measures (35, 36). The continuous rise in the burden of NALC in the BRICS countries indicates that NASH is gradually becoming one of the important etiological factors of HCC (37). Its epidemiological transition is influenced by multiple factors, including socioeconomic conditions, the level of public health interventions, and healthcare resource allocation.

Sex-stratified analyses demonstrated that NALC ASIR exhibited a marked increase in men relative to women over the past three decades. Brazil, the Russian Federation, China, and South Africa exhibited the most significant performance in this regard, reflecting the broader impact of sex disparities on the epidemiology of HCC on a global scale (38). Socioeconomic determinants may play a substantial role in shaping sex disparities within BRICS nations. Men often have greater occupational exposure to hepatotoxic agents and a higher prevalence of behavioral risk factors. In contrast, women may face structural barriers to timely screening, vaccination, and treatment due to gender-specific differences in healthcare access and health-seeking behavior (39, 40). Research has indicated that male patients suffering from NASH (non-alcoholic steatohepatitis) exhibit a more rapid rate of pathological progression due to a higher fat load and more severe metabolic abnormalities (41). Moreover, androgens have been demonstrated to potentially augment the likelihood of developing HCC through their capacity to expedite the progression of fat accumulation and hepatic fibrosis (42). Conversely, estrogens are believed to exert a protective effect (42). India constitutes a comparatively exceptional case, in which the disparities in IR between men and women are less pronounced than in other countries. This phenomenon may be attributable to the nation’s distinctive cultural milieu, dietary patterns, and sex role dynamics (41).

With regard to temporal trends, the IR of NALC in the BRICS countries has generally exhibited significant increases of varying degrees since 2000. Among the country-specific changes, the IR in South Africa and China remained at a high level throughout the study period. This phenomenon is indicative of the multiple public health transitions experienced by both countries during that period. These transitions encompass changes in dietary structure, rising obesity rates, and increasing prevalence of diabetes mellitus and metabolic syndrome (43). The aforementioned transitions have been accompanied by accelerated economic development and urbanization, which have significantly elevated the risk of NALC (44). Especially in China, the advent of sophisticated medical technology and the enhancement of public awareness with regard to screening have collectively resulted in a greater number of cases of NALC being detected at an early stage (45). Recent data indicates a gradual yet consistent upward trend in India IR, with overall levels remaining below those observed in China and South Africa. The aforementioned factors may be related to the following: the population structure of the area being youthful, the relatively low burden of metabolic diseases, and the limited capacity for disease identification and management (46). Brazil IR has exhibited a lower degree of variability and has remained at a comparatively low level for a protracted period, which may be ascribed to the country’s relatively mature universal health insurance system and its policies for the management of chronic diseases (31).

An analysis of the age distribution revealed that the IR of NALC in the BRICS countries exhibited an increase with age from 1990 to 2021, reaching its peak among individuals aged 80 to 90, after which it underwent a slight decline. This trend is consistent with the overall changes in cancer epidemiology that have occurred in the context of global population ageing (3). Firstly, research has demonstrated that older adults exhibit a higher susceptibility to liver cancer in comparison to younger adults (47). Secondly, the likelihood of NASH progressing to HCC rises markedly with age, a phenomenon that is predominantly ascribed to the accumulation of metabolic disorders, including chronic fat deposition and insulin resistance (48). Furthermore, the likelihood of developing HCC in the elderly is augmented by a decline in immune function, a diminution in hepatic metabolic rate and regeneration, and the presence of co-morbidities such as diabetes mellitus, hypertension, and other prevalent chronic diseases (49, 50). The combination of multiple risk factors results in an increased morbidity burden of NASH-associated HCC in the elderly population and should be prioritized as a target for screening and prevention in future public health interventions.

Birth cohort analyses have demonstrated that, in conjunction with global economic development and dietary shifts, later-born populations are predisposed to a heightened risk of developing NALC. In particular, a consistent upward trend in NAFLD-related risk was observed in cohorts born after 2000–2007. This trend may be indicative of a combination of multiple factors, including but not limited to the global obesity epidemic, decreased physical activity, increased mental stress, unhealthy eating habits, and the increased prevalence of metabolic syndrome (51). As a consequence of exposure to a range of adverse factors, including but not limited to widespread consumption of ultra-processed foods and sugar-rich beverages, economic constraints, and adverse childhood experiences, younger generations are more susceptible than earlier birth cohorts to the development of metabolic risk factors in early adulthood (52). This birth cohort trend indicates a necessity for the implementation of preventive intervention strategies in adolescents and young adults at an earlier stage. Such strategies may include the improvement of dietary composition, the promotion of physical activity, and the enhancement of obesity and metabolic risk management. The implementation of these strategies has the potential to reduce the future burden of NALC and to optimize the allocation of health resources.

In accordance with the findings of the future trend prediction model, it is anticipated that the disease burden of NALC in the BRICS countries will endure for a period of 15 years, with an escalating trend being observed in certain countries. It is projected that ASIR in South Africa will continue to rise, with the most significant increase being closely related to the level of socio-economic development, lifestyle changes, and insufficient capacity for public health interventions (36). In contrast, the remaining four BRICS countries (China, India, Brazil, and the Russian Federation) have demonstrated a stabilization of their NALC incidence projections (38, 53). In this context, the key to curbing the continued growth of NASH-associated hepatocellular carcinoma lies in strengthening early screening and metabolic risk factor interventions in high-risk populations and promoting the popularization of healthy lifestyle and systematic management of metabolic diseases (54).

Further analysis suggests that epigenetic regulation, such as DNA methylation, histone modification and microRNA dysregulation, may accelerate the progression of NAFLD to NASH, an important precursor state of hepatocellular carcinoma (55). Epigenetic differences, lifestyle, and environmental exposure variations among populations in different regions may explain the inconsistencies in NASH and related hepatocellular carcinoma burden observed in the BRICS countries (56, 57). The BRICS countries are distinguished by considerable rural–urban and socioeconomic disparities, which have a substantial impact on disease screening and chronic disease management (58). Inadequate facilities, medicines, and human resources in rural and marginalized areas prevent the timely identification of underlying infections and hinder the continuity of chronic disease management, creating “service gaps” in policy implementation and exacerbating health inequalities (39, 59). Notably, the recently proposed age, period, and cohort model optimization method for sparse cancer data provides a key methodological reference for interpreting age- and cohort-specific trends in countries with a low incidence of cancer (60). A multidimensional perspective integrating molecular biological mechanisms, health system differences, and modeling approaches is essential to comprehensively understand the differences in the burden of NALC in the BRICS countries.

## 5 Limitations

The present study is not without its limitations. Firstly, in areas of high alcohol consumption, there are challenges in distinguishing NASH-related HCC from alcohol-related HCC, which may lead to a bias in the classification of some cases, thus affecting the interpretation of the results. Secondly, the NCI APC web tool does not adjust for data sparsity in extreme age groups (≥90 years) where case counts are often low, potentially leading to unstable rate estimates and wide confidence intervals. Thirdly, the ARIMA model struggles to accurately predict the potential impact of new interventions, public health policy changes, and advances in medical diagnostic or therapeutic technologies on future morbidity. Finally, the GBD model is principally predicated on the burden of disease statistics and has not yet fully incorporated variables such as healthcare resources, health system construction, and policy interventions, which may have a substantial impact on the incidence of NALC. Consequently, the applicability and representativeness of the unified model across countries may be constrained, necessitating caution during the interpretation of results.

## 6 Conclusion

The present study reveals a sustained upward trend in NALC incidence in the BRICS countries during 1990–2021 and significant differences in the pace and drivers of growth across countries. The heterogeneity reflected by the joinpoint and APC models reveals gaps in the burden of metabolic diseases, public health response, and policy implementation across countries. In order to address this challenge, priority should be given to the promotion of metabolic disease-related early screening, health behavior interventions, and systematic management—for instance, South Africa should consider ways to enhance liver cancer screening among high-risk demographics. In contrast, China should prioritize the comprehensive prevention and management of metabolic diseases to facilitate precise interventions and optimize resource utilization, thereby enhancing the public health translational value of research.

## Data Availability

The original contributions presented in the study are included in the article/supplementary material. Further inquiries can be directed to the corresponding author.
